# Herpes Zoster Encephalitis

**Published:** 1954

**Authors:** R. D. G. Creery

**Affiliations:** Principal Paediatric Registrar, Royal Belfast Hospital for Sick Children; Late Medical Registrar, Southmead Hospital, Bristol.


### HERPES ZOSTER ENCEPHALITIS
### in association with the Ramsay Hunt syndrome

*Report of a case*

BY

R. D. G. CREERY, M.D., M.R.C.P., D.C.H.

*Principal Paediatric Registrar, Royal Belfast Hospital for Sick Children; late Medical Registrar, Southmead Hospital, Bristol.*

Encephalitis is one of the rarer complications of herpes zoster. The clinical picture of the encephalitis may be fulminating with a fatal outcome, or it may be of a much milder nature with ultimate recovery, as in the case here recorded. The present case is reported as an example of its association with another occasional manifestation, the Ramsay Hunt syndrome, or so-called " geniculate herpes " (Hunt, 1907). This syndrome in its most severe form, comprises peripheral facial palsy, herpetic eruption on the ear, face or neck, and involvement of the auditory nerve.

### CASE REPORT

A carpenter, aged forty years, was admitted to hospital on 25th May, 1951. Several weeks before, he had been in contact with a case of " shingles ". One week before admission, he complained of pain in the right ear. Two days later he had difficulty with speech and noticed right-sided facial weakness. Later he became " dizzy " and complained of severe headache and photophobia. On the day before admission his right ear began to discharge, and he felt drowsy. Examination showed an apprehensive man, fully conscious, co-operative and apyrexial. The relevant findings were as follows: complete right facial paralysis (lower motor neurone in type), with loss of taste on the anterior part of the tongue; right-sided deafness (middle-ear in type, as judged by tuning-fork tests). Cold caloric tests were normal. The right ear showed an external otitis with herpetic vesicles in the external auditory meatus and on the pinna. The tympanic membrane was involved, but was not bulging, nor was there any perforation. When standing, with eyes open or closed, he tended to fall to the right. There was no other clinical abnormality. Two days after admission he suddenly became rigid, and lost consciousness for several minutes. He rapidly recovered, but remained drowsy for some hours. A transient weakness of the right external rectus muscle was noted. The plantar reflexes were equivocal, later becoming flexor. Lumbar puncture was performed. The pressure was 260 mm. of cerebrospinal fluid and the fluid was faintly cloudy. The cerebrospinal fluid contained 1[illegible] leucocytes c.mm., the majority being polymorphonuclears; protein 130 mgm. per cent.; chlorides 730 mgm. per cent.; sugar 54 mgm. per cent. There were no organisms and the fluid was sterile on culture. He was seen the following day by Dr. A. M. G. Campbell, who felt that this was a case of herpes zoster encephalitis, presenting as the Ramsay Hunt syndrome. From then on, the patient's condition gradually improved, the facial weakness decreased, the herpetic lesions healed, and he was discharged on 19th June, 1951. When last seen as an out-patient three months after admission, he was well and had only a slight facial weakness and a minor degree of deafness.

### DISCUSSION

The association of encephalitis with zoster infection was discussed by Schiff and Brain (1930), who reviewed the literature, and recorded a further fatal case

They concluded that, although in some patients the zoster was a complication of an independent encephalitis, in others an extension of the zoster infection had produced the encephalitic picture. In their case this latter conclusion was supported by the results of cerebrospinal fluid complement-fixation tests. Biggart and Fisher (1938), in their report of a fatal case of meningo-encephalitis complicating herpes zoster, gave a very full account of the clinical and pathological findings. The patient became drowsy five weeks after the onset of herpes zoster in the distribution of the right ilio-inguinal nerve, and later became comatose and delirious. The cerebrospinal fluid contained 20 cells per cmm. Pathologically, there were dorsal ganglionitis, mononuclear infiltration of the subarachnoid space, posterior myelitis, and encephalitic changes such as peri-vascular round-cell infiltration. They concluded that these findings supported the possibility that the encephalitis was due to zoster infection, as the lesions in the medulla and mid-brain appeared to be merely an upward extension of the same lesions found in the cord. The encephalitic picture in the present case comprised drowsiness and headache, followed by a seizure with transient coma and sixth-nerve paresis. The cerebrospinal fluid pressure was high, as in Schiff and Brain's case. An interesting feature was the high proportion of polymorphonuclears in the fluid which probably indicated an early stage of the reaction; mononuclear cells are stated to be more commonly found (Brain, 1951). Unfortunately no virus studies were undertaken in this case. Denny-Brown *et al.* (1944) cast considerable doubt on the concept of geniculate ganglionitis as the cause of the Ramsay Hunt syndrome. In a fatal case which had presented this syndrome, they were unable to show any changes within the geniculate ganglion. They found, however, a definite motor neuritis of the facial nerve. On the other hand, Parkinson (1948) agreed that the primary lesion is in the geniculate ganglion. Critchley (1947) surveyed the present rather confused position of this syndrome, and concluded that until a better term is forthcoming, based upon exact knowledge of the morbid anatomy, the " Ramsay Hunt syndrome " may conveniently be used to describe all cases where facial paralysis is associated with an outbreak of herpes zoster vesicles anywhere upon the head and neck. It is probable that the syndrome cannot be explained on any one pathological lesion, but that it is a further example of the pleomorphism which characterizes zoster infection: at one time there may be a ganglionitis, at another encephalitis, or again, a motor neuritis, or, as is probable in the present case, a combination of all three. The local eccentricities in the features of the syndrome could be explained by postulating a varying attack on the nerves and their ganglia supplying the region of the head and neck. It is suggested that the term " geniculate herpes " might be dropped, as it does not satisfactorily explain the variations in the clinical picture and has little, if any, pathological foundation. Recent advances in the use of antibiotics have produced hopes that some of these drugs might be helpful in the treatment of herpes zoster. Binder and Stubbs (1949), and Finland *et al.* (1949), reported good results with aureomycin. It is to be noted that neither series was controlled. More recent work, in which controls were used, by Carter (1951), and Kass *et al.* (1952), suggests that there is little evidence in favour of a specific effect against the zoster virus by either aureomycin or chloramphenicol. Carter did suggest, however, that secondary infection was avoided, but Kass *et al.* were unable to find any significant difference between treated and untreated cases. That the treatment of zoster infectio with antibiotics is not satisfactory is suggested by the experience of Foster an Jackson (1951) who reported the occurrence of herpes zoster encephalitis in patient treated with aureomycin for the original infection. At present it probably justifiable to use these antibiotics only in severe cases, such as ophtha mic zoster, or where a potentially fatal complication such as meningo-encephaliti has occurred.

### SUMMARY

A case of herpes zoster presenting with auricular eruption, facial paralysis an encephalitis is reported. The pleomorphism of the disease is noted. Herpeti encephalitis and the Ramsay Hunt syndrome are discussed. The use of th term " geniculate herpes " is deprecated. It is suggested that treatment wi antibiotics might be reserved for severe cases and for those with serious compl cations.

I wish to thank Dr. A. M. G. Campbell for his opinion, Dr. F. J. W. Lewis for th pathological report and Dr. J. A. Birrell for permission to publish.

### REFERENCES

Biggart, J. H., and Fisher, J. A. (1938). *Lancet*, **2,** 944.

Binder, M. L., and Stubbs, L. E. (1949). *J. Amer. med. Ass.*, **141,** 1050.

Brain, W. R. (1951). *Diseases of the Nervous System*, 4th ed., 491. London.

Carter, A. B. (1951). *Brit. med. J.*, **i,** 987.

Critchley, M. (1947), in *The Medical Annual*, 281. Bristol.

Denny-Brown, D., Adams, R. D., and Fitzgerald, P. J., (1944). *Arch. Neurol. a Psychiat.*, **51,** 216.

Finland, M., Finnarty, E. F., Jr., Collins, H. S., Baird, J. W., Gocke, T. M., and Kas E. H., (1949). *New. Engl. J. Med.*, **241,** 1037.

Foster, J. H., and Jackson, A. H. (1951). *Conn. med. J.*, **15,** 199, quoted in *Excerpt Medica* (Internal Medicine), (1951), **5,** 1482. Amsterdam.

Hunt, J. Ramsay (1907). *J. nerv. ment. Dis.*, **34,** 73.

Kass, E. H., Aycock, R. R., and Finland, M. (1952). *New Engl. J. Med.*, **246,** 167.

Parkinson, T. (1948). *Brit. med. J.*, **1,** 8.

Schiff, C. I., and Brain, W. R. (1930). *Lancet*, **2,** 70.

